# A Closer Look into Autoimmune Liver Diseases

**DOI:** 10.3390/ijms26051863

**Published:** 2025-02-21

**Authors:** Branka Filipovic, Marija Marjanovic-Haljilji, Dragana Blagojevic, Milica Dragovic, Emilija Krsmanovic, Ana Matovic, Natasa Panic, Stanimir Kiurski, Zagor Zagorac, Miljan Milanovic, Olivera Markovic, Aleksandra Djokovic, Tijana Glisic, Sanja Dragasevic, Dusan Popovic

**Affiliations:** 1 Department of Gastroenterology, Clinical and Hospital Center “Dr Dragisa Misovic-Dedinje”, Heroja Milana Tepica 1, 11020 Belgrade, Serbia; branka.filipovic3@gmail.com (B.F.); n.blagojevic0205@gmail.com (D.B.); milicadragovic50@gmail.com (M.D.); emilija.krsmanovic97@gmail.com (E.K.); filipovicn@live.com (N.P.); stanimir.kiurski@gmail.com (S.K.); dr.dusan.popovic@gmail.com (D.P.); 2 Faculty of Medicine, University of Belgrade, Dr Subotica Starijeg 8, 11000 Belgrade, Serbia; drmiljanmilanovic@gmail.com (M.M.); markovic.olivera@bkosa.edu.rs (O.M.); drsaska@yahoo.com (A.D.); tijana.glisic78@gmail.com (T.G.); dragasevicsanja@gmail.com (S.D.); 3 Department of Cardiology, Clinical and Hospital Center “Dr Dragisa Misovic-Dedinje”, Heroja Milana Tepica 1, 11020 Belgrade, Serbia; anamatovic1998@gmail.com; 4 Clinic for Surgery, Clinical and Hospital Center “Dr Dragisa Misovic-Dedinje”, Heroja Milana Tepica 1, 11020 Belgrade, Serbia; drzzagorac@gmail.com; 5 Department of Hematology, Clinical and Hospital Center “Bezanijska Kosa”, Dr Zorza Matea s/n, 11080 Belgrade, Serbia; 6 Department of Cardiology, Clinical and Hospital Center “Bezanijska Kosa”, Dr Zorza Matea s/n, 11080 Belgrade, Serbia; 7 Clinic for Gastroenterology and Hepatology, University Clinical Centre of Serbia, 11000 Belgrade, Serbia

**Keywords:** autoimmune liver disease, hepatitis, primary sclerosing cholangitis, primary biliary cholangitis

## Abstract

Autoimmune liver diseases involve a heterogeneous group of chronic inflammatory disorders, including autoimmune hepatitis, primary biliary cholangitis, and primary sclerosing cholangitis. Sometimes presented consistently as an overlapping syndrome, their pathogenesis is rather complex and has yet to be fully elucidated, despite extensive research efforts. This review article corroborates the molecular mechanisms of autoimmune liver diseases, as well as existing and potential therapeutic modalities.

## 1. Introduction

Autoimmune liver diseases (AILDs) encompass a heterogeneous group of chronic inflammatory disorders, including autoimmune hepatitis (AIH), primary biliary cholangitis (PBC), and primary sclerosing cholangitis (PSC) [1]. These conditions may present concurrently as an overlapping syndrome, which is characterized by the symptoms of two or more diseases [2,3,4]. The prevalence of AILD is increasing, with an annual incidence of 1–2 cases per 100,000 people for each condition [5].

The pathogenesis of autoimmune liver diseases has yet to be fully defined, but it is believed to involve complex interactions between genetic predisposition, immune responses, and environmental factors [6,7]. Evidence of genetic predisposition includes familial clustering, high concordance rates in monozygotic twins, and an increased disease risk among first-degree relatives compared to the general population [8,9]. In all three diseases, liver damage is mediated by an immune response marked by an imbalance between effector and regulatory T lymphocytes, which results in the activation of cytotoxic T-cells [6,10,11]. Autoimmune reactions may be initiated by molecular mimicry, wherein microbiological peptides resembling autoantigens lead to the misrecognition of self-antigens [12,13]. Although autoimmune liver diseases have common etiological factors, they target different liver cell populations. While autoimmune hepatitis primarily affects hepatocytes, primary biliary cholangitis and primary sclerosing cholangitis predominantly target the epithelial cells of small interlobular and larger intra- and extrahepatic bile ducts [12]. Primary biliary cholangitis and primary sclerosing cholangitis are generally resistant to immunosuppressive therapy, unlike AIH, which responds well to such treatment [7]. Notably, autoimmune hepatitis and primary biliary cholangitis (PBC) are more prevalent in middle-aged women, whereas primary sclerosing cholangitis is more common in men, and is characterized by a high rate of progression to cirrhosis or hepatocellular carcinoma [14,15,16,17].

Unfortunately, there are currently no therapies which target the key pathophysiological processes in the pathogenesis of these diseases that allow for a complete cure, and such treatments often result in progression to an extent that necessitates liver transplantation [18,19,20,21]. A better understanding of the etiopathogenesis of these conditions could enhance diagnostic and treatment methods, thereby reducing mortality rates and the need for liver transplantation [22]. In summary, this paper will provide a detailed examination of the etiopathogenesis and molecular mechanisms of each disease, as well as their clinical presentation and the currently available therapeutic options. We believe that the insights gained will lead to new opportunities for clinical research.

## 2. Autoimmune Hepatitis

Hepatocellular necrosis and inflammation are the hallmarks of autoimmune hepatitis (AIH), a chronic, progressive immune- mediated liver disease that can lead to fibrosis and, ultimately, cirrhosis. It is a multifactorial illness, with a prevalence three times higher in females than in males, often followed by other autoimmune disorders such as vitiligo, insulin-dependent diabetes, nephrotic syndrome, urticaria pigmentosa, hemolytic anemia, idiopathic thrombocytopenia, and celiac disease [23].

A diagnosis of AIH can be confirmed according to certain criteria: female preponderance, elevated levels of immunoglobulin G (IgG), the presence of autoantibodies, and pathohistological verification suggesting interface hepatitis—laboratory findings that suggest fluctuations in a patient’s aminotransferase level. Since there must be more than one diagnostic test to confirm AIH, the International Autoimmune Hepatitis Group (IAIHG) has created a scoring system (the original one in 1999 and the revised one in 2008), where the important role of serum antibodies has been described. In the original one, scores range from 0 to +3, based on the ANA, SMA or anti-LKM1 titer, where 0 is a titer below 1:40, +1 is given for a titer of 1:40, +2 is given for a titer of 1:80 and +3 points are given if the titer is above 1:80. This scoring system also includes other antibodies such as Anti-SLA/LP, actin, ASGPR, pANNA and other diagnostic criteria like the ALP:AST ratio, serum immunoglobulins, histology findings, viral markers of active infection, hepatotoxic drug history, alcohol consumption, other immune diseases, human leukocyte antigen and response to therapy. A pretreatment score > 15 indicates definite AIH, and a pretreatment score of 10 to 15 indicates probable AIH. A posttreatment score > 17 indicates definite AIH, and a posttreatment score of 12 to 17 indicates probable AIH. According to the revised scoring system, a titer of ANA or SMA above 1:40 is worth 1 point; a titer above 1:80 or anti-LKM1 above 1:40 or SLA-positive status is worth 2 points. A score ≥ 6 indicates probable AIH; a score ≥ 7 indicates definite AIH [24]. AIH can be classified into two categories based on the type of serum autoantibodies present: one type is positive for liver kidney microsomal antibody type 1 (LKM-1) (type 2 AIH, AIH-2) and another type is positive for smooth muscle antibody (SMA) and/or antinuclear antibody (ANA) (type 1 AIH, AIH-1) [25].

There are significant differences in the style of presentation and the age of onset between these two; for example, it is common for AIH-2 to have an earlier age of onset, to present with fulminant hepatic failure—defined by the presence of an international normalized ratio (INR) > 1.5–2 and encephalopathy—and to be connected with IgA deficiency, while AIH-1 is associated with a more subtle beginning, with symptoms such as malaise, fatigue and arthralgia; however, it should be mentioned that a significant number of patients are asymptomatic and only exhibit increased transaminase levels after biochemical screening [26,27,28].

**Figure 1:**
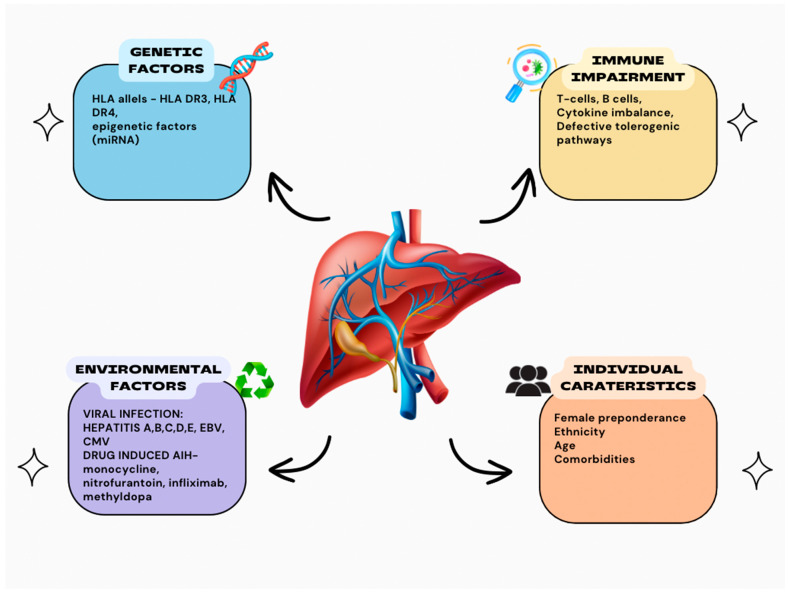
Pathogenesis of AIH. Abbreviations: HLA—human leukocyte antigen; EBV—Epstein–Barr virus; CMV—cytomegalovirus.

It is believed that the interaction between molecular mimicry, impaired immunoregulatory mechanisms, and environmental and individual factors forms the basis of AIH pathogenesis (Figure 1).

## 3. Genetics

Genome-wide association studies (GWASs) have identified single-nucleotide polymorphisms (SNPs) associated with AIH, such as HLA alleles HLA DR3 and HLA DR4 [29].

Although genetic polymorphisms vary between different ethnic groups, the HLA loci responsible for AIH-1 susceptibility are associated with the HLA-DRB1 variant in chromosome 6, representing a class II MHC. DRB1*0301 and DRB1*0401 are the primary susceptibility alleles for type 1 AIH in North America and northern Europe, where a high correlation has been found between type 1 AIH and HLA A1-B8-DR3 and HLA DR4 [30,31]. Muratori et al. distinguish HLA DR11 as a geographically specific protective factor against type 1 AIH, and B8-DR3-DQ2 as the prevalent phenotype of type 1 AIH in Italy as well [32].

HLA-DR3 (DRB1*0301) and -DR4 (DRB1*0401) molecules are found in North American and European populations and the -DR4 (DRB1*0401) allele is found in the Japanese population [29,30]. In a separate study, patients without HLA-DR3 or HLA-DR4 were more commonly found to have HLA-DR13 or HLA-DR7, and their clinical and laboratory profiles were similar to those of patients with HLA-DR3.

As far as AIH-2 is concerned, AIH-2 is usually associated with predisposing DRB1*0701 and DRB1*0301. In a different study, Canadian and French Caucasian children presented the DQB1*02:01 allele as the main genetic determinant of susceptibility [33]. It has been stated that autoimmune polyendocrinopathy–candidiasis–ectodermal dystrophy (APECED) syndrome can be connected to AIH-2 through HLA-DQB1*0301 and DQB1*0201 alleles [23].

There have been several reports on the effect of miRNA on the pathogenesis of many liver diseases such as non-alcoholic steatohepatitis (NASH), hepatocellular carcinoma (HCC), hepatitis C virus (HCV) and AILD. It is believed that miRNA regulate not only inflammation but also cell apoptosis, necrosis, proliferation, lipide and glucose accumulation and liver fibrosis and, furthermore, cirrhosis. MiR-155 levels increase in Kupffer cells after alcohol feeding, and tumor necrosis factor (TNF) has been identified as a target of miR-155 that promotes liver inflammation [34].

Upon sustaining hepatic fibrogenic damage, hepatic stellate cells (HSCs) proliferate and differentiate into myofibroblasts-like cells. Numerous miRNAs have been found to be involved in controlling HSC activation. Serum levels of miR-542, miR-652, and miR-181b are reduced in cirrhosis, while increased levels of miR-571 have been suggested as a possible biomarker of liver fibrosis [35].

## 4. Immune Dysregulation

The development and occurrence of immune-mediated hepatitis are significantly influenced by Th17 and Treg cells. There are three subtypes of Treg cells: CD4+, CD25+ and CD8+. CD4+ T-cells play a vital role in regulating B-cell antibody production, influencing the cytotoxic activity of CD8+ T-cells, overseeing phagocytic processes, and modulating cellular movement. An essential part of preserving cell homeostasis is carried out by CD4+ CD25+ Foxp3+ Treg inhibitory effector cells. Patients with AIH exhibit diminished Tregs and low expression of FOXP3. The FOXP3 gene encodes a DNA-binding protein FOXP3, which plays an important role in the activation and regulation of CD4+CD25+ regulatory T lymphocytes. The dysregulation of this pathway induces autoimmunity.

Another important cell subtype is CD8+, which is also activated in AIH. This type of cells recognizes and kills hepatocytes that express abnormal antigens or self-antigens that mimic viral proteins, which represent molecular mimicry [36].

B-cells are essential in the production of autoantibodies in AIH. These autoantibodies bind to specific liver antigens and can form immune complexes. This triggers the activation of the complement system, leading to tissue damage, inflammation and further fibrosis. Recent research suggests that alterations in B-cell subsets, particularly an increase in memory B-cells, are linked to the severity and activity of the disease in individuals with AIH. The role of B-cells extends to cytokine production and antigen presentation. Examples of B-cell-derived cytokines include TNF-α and chemokine (C-C) ligand 3(CCL3), which control Th1 cell responses; IL-2, which stimulates Th2 memory responses; and interferon γ (IFNγ), which stimulates Th1 responses [37].

Another important pathophysiological mechanism is the imbalance between pro- and anti-inflammatory cytokines such as elevated levels of TNF-α and interleukin-6 (IL-6). These cytokines promote the activation of HSCs and contribute to liver inflammation and fibrosis [23].

## 5. Environmental Factors

The clearest evidence of an environmental role in the development of AIH comes from viral infections and drug exposures. Numerous viruses, such as Epstein–Barr (EBV), Varicella Zoster (VZV), and hepatitis A, B, C, and E, and also Sars-Cov-2, have been connected to AIH. Although only a few studies have been conducted regarding the direct impact of SARS-CoV-2 on the liver, it is believed that several SARS-CoV-2 components share structural similarities with human proteins [11], which allow immune responses triggered against the virus to cross-react with self-proteins. This phenomenon is known as molecular mimicry. These elements could trigger autoimmunity by causing the formation of several autoantibodies [38].

A report from various regions in Italy found that 10 patients with AIH who were receiving immunosuppressive treatment had a clinical course of COVID-19 similar to that of patients who were not on immunosuppressants. Additionally, telephone surveys conducted in Northern Italy did not show a higher COVID-19 mortality rate among patients with AIH [39].

Drug exposures have also been identified as causal triggers for AIH (drug-induced AIH [DI-AIH]), with nitrofurantoin and minocycline being the most common catalysts. These drug exposures frequently exhibit clinical autoimmune characteristics. There have also been reports on hepatotoxicity of oxyphenisatine, methyldopa, diclofenac, interferon, atorvastatin and biologic agents such as infliximab, natalizumab, and adalimumab [40].

## 6. Therapy for Autoimmune Hepatitis

The overall goal of AIH treatment is to induce clinical and biochemical remission and prevent further progression of the disease, where clinical remission means an absence of symptoms and biochemical remission—normalization of AST, ALT and IgG levels [41].

Also, performing a liver biopsy before discontinuing therapy is the preferred approach. In adult patients, whether or not a pre-withdrawal liver biopsy was performed, the relapse rates after at least 2 years of treatment were similar (30% vs. 21%, *p* = 0.57), with serum AST and ALT levels being either normal or near-normal during that time. Among 28 patients with AIH who had been in biochemical remission for at least 2 years before stopping treatment, 15 (54%) stayed in remission during a median follow-up of 28 months (range: 17–57 months). These patients had an ALT level < 50% of the upper limit of normal (ULN) and normal serum IgG levels < 1200 mg/dL. Of the 13 patients who had a liver biopsy before discontinuing treatment, 11 had normal liver test results and normal liver tissue, and 46% of them relapsed later. These results suggest that sustained normal liver tests during treatment may provide better outcome predictions than liver tissue examination. However, a pre-withdrawal liver biopsy is still strongly recommended for children to confirm the resolution of inflammation [42].

According to the recommendations stated by the American Association for the Study of Liver Diseases (AASLD) in 2019, standard induction therapy in AIH includes a combination of high-dose prednisolone or prednisone with or without azathioprine (AZA). It is stated that the recommended dose of prednisolone is 40–60 mg daily as a monotherapy or 20–40 mg in combination with AZA. The typical starting dose of AZA is 50–100 mg daily in adults and 1–2 mg/kg daily in children. The dose of AZA should be adjusted so that side effects and the toxicity of its metabolites are avoided.

The dose of prednisone or prednisolone should be gradually reduced to 10 mg or to a dose that maintains remission, informed by laboratory findings obtained every 2 weeks. Monotherapy with corticosteroids is reserved for situations in which the duration of treatment is expected to be less than 6 months, or where AZA is contraindicated, as stated in the guidelines. After inducing and maintaining remission, the main goal is to exclude corticosteroid therapy and only continue with the immunosuppressive therapy. Treatment is continued for at least 2 years. The most common side effects of corticosteroid therapy include osteoporosis, diabetes mellitus (DM), hypertension, glaucoma, depression, anxiety, weight gain. Side effects of AZA include cytopenia, severe leucopenia or bone narrow suppression, cholestatic liver damage, nonmelanoma skin cancer.

Second-line therapy is recommended for patients who exhibit an incomplete response, have severe side effects, and are intolerant to first-line treatment. As an alternative to prednisone/prednisolone, synthetic corticosteroid budesonide can be used and is proven to be effective because of its 90% first-pass hepatic clearance rate. It has less systemic side effects but because of its inability to reach the liver with portal hypertensive shunts and its promotion of portal vein thrombosis, it should not be prescribed to patients with cirrhosis and acute severe AIH [43].

For patients with intolerable AZA side effects, alternative therapies include mycophenolate mofetil (MMF)- inosine monophosphate dehydrogenase inhibitor or calcineurin inhibitors such as tacrolimus and ciclosporin A. Their mechanism of action consists of inhibiting calcineurin, which subsequently inhibits the transcription of IL-2 and other proinflammatory cytokines and disrupts T-cell activation (T suppressor and T cytotoxic cells dominantly) [44]. Methotrexate, infliximab, rituximab, sirolimus, and everolimus are used as salvage therapy only in a small number of patients [44].

## 7. Primary Biliary Cholangitis

Primary biliary cholangitis (PBC), previously referred to as primary biliary cirrhosis, is the most common type of presumed AILD with a chronic course. It is characterized by progressive damage to the small intrahepatic bile duct (diameter < 100 µm) that results in inflammation and cholestasis and, consequently, leads to cirrhosis, with all of its complications. PBC is a disease that occurs primarily in Caucasian women aged between 40 and 60 years, although current data show a higher prevalence in males than previously described [45]. As one of the main progressive cholangiopathies, PBC is subject to ongoing research, especially because its etiopathogenesis is still mostly unknown. It is considered that mutual interaction between genetics and environmental factors in a sensitive person plays a key role [46].

## 8. Genetics and Epigenetics

Genome-wide association studies (GWASs) indicate familial occurrence and a high concordance rate among monozygotic twins as the main indicators of genetic inheritance [47]. HLA genes play an essential role in numerous infectious and autoimmune diseases, including AIH [48]. Meta-analysis carried out by Li et al. identified relations between HLA class II and disease susceptibility to PBC and indicated that HLA DR*07 and *08 alleles were risk factors for PBC in certain populations, while DR*11, *12, *13, and *15 alleles were protective factors [49]. The fact that some of these alleles were found in individuals with hepatitis C, hepatitis B and human papilloma virus infection speaks in support of molecular mimicry as an initiator of autoimmune reactions in PBC [50,51,52]. In addition, data from numerous studies indicate that 80–90% PBC patients do not carry the most common HLA susceptibility alleles. GWASs have identified more than 40 non-HLA PBC-predisposition loci at a genome-wide level of significance [53]. From early indications of somatic mosaicism to the more current and reliable data resulting from a considerable meta-analysis, the set of evidence demonstrating the function of chromosome X in PBC has kept expanding, as one of the explanations of female predominance [54]. There are more indicators that show the role of epigenetics modifications, including DNA methylation and histone modification, in PBC pathogenesis [55]. Hirschfield et al. have demonstrated a relationship between PBC and common genetic variants at IL-12 as one of the proinflammatory cytokines mainly responsible for CD4+T-cells’ differentiation into Th1 cells. Furthermore, IL-12 deficiency was noticed in several pediatric cases who developed PBC [55,56]. Genetic studies have brought attention to the role of immune modulation in the pathophysiology of PBC and have revealed abnormal pathways that contribute to the disease. These pathways include antigen presentation, T and myeloid cell development, and B-cell activity.

## 9. Immunology

High serum AMA titers and autoreactive T- and B-cell responses against mitochondrial self-antigens are among the evidence suggesting that this is an autoimmune condition. AMA is a serological signature of PBC, as approximately 95% of PBC patients have positive results for AMA. In addition, the development of PBC is typically indicated by the presence of AMA before any clinical symptoms manifest. Consequently, AMA is regarded as a highly specific and sensitive biochemical marker for PBC [57]. The E2 component of the pyruvate dehydrogenase complex (PDC), in particular, is one of the enzymes of the 2-oxoacid dehydrogenase family that AMA identifies as having liposylated domains. A specific lack of tolerance to the E2 subunit of the mitochondrial PDC (PDC-E2) leads to dysregulation of the innate and acquired part of the immune system and a focused immunological response against biliary epithelial cells (BECs) [58]. Research has demonstrated that PDC-E2, the 2-oxo-glutarate dehydrogenase complex (OGDC-E2), and the branched-chain 2-oxoacid dehydrogenase complex (BCOADC-E2) are still antigenically active within antibodies of the apoptotic BECs in PBC patients. This is primarily because of a defect in the BECs’ ability to clear antigenic proteins, while other cells that do not have altered autoantigen expression levels do not retain the autoantigen epitopes that the AMA in PBC patients can recognize. Depletion of PDC-E2’s cellular glutathionylation is hypothesized to have a role in maintaining autoantibody recognition [59]. However, a percentage of PBC patients may not test positive for AMA even when the most sensitive AMA tests are employed, despite some research suggesting that seronegative cases may eventually become AMA-seropositive. It suggests that alternative non-mitochondrial antigen-driven processes may be at play in PBC pathogenesis, which emphasizes the significance of correctly identifying and regularly using diagnostic surrogate indicators that can aid in the diagnosis of PBC in AMA-negative cases [60]. Antinuclear antibodies (ANAs), especially anti-Sp100 and gp 210, are detected in 30–50% of PBC patients and can be significant for diagnosis and prognosis. Since 2017, anti-gp210, which targets one of the constitutive proteins od the nuclear pore complex, has emerged as a diagnostic marker for AMA-negative PBC, increasing the diagnostic rate and preventing some patients from undergoing needless liver biopsies [61]. Anti-gp210 can be an indicator of prognosis in addition to being a highly specific (98%) antibody for PBC diagnosis. Compared to anti-gp210-negative individuals, anti-gp210-positive PBC patients had worse prognoses, higher levels of biochemical liver tests, more severe histological presentations, and worse responses to ursodeoxycholic acid (UDCA) (39.3% vs. 16.7%) [62]. The pathogenesis, although still unclear, underpins the primary theory of bacterial molecular mimicry, which requires more research. For the first time, Granito et al. discovered Sp140 as a unique, highly specific autoantigen in PBC, which was highlighted in a study by Saare et al. [63]. The highly common presence of anti-Sp140 and anti-Sp100 antibodies raises the diagnostic relevance of these reactivities, which are especially helpful in AMA-negative individuals, alongside some other new biomarkers, such as anti-kelch-like-12 and anti-hexokinase-1 antibodies [64,65].

It is important to point out that the disease affects the small and medium intrahepatic bile ducts, while the large intra- or extrahepatic bile ducts are spared. The reason why PBC does not target uniformly is still unknown, but some evidence is in favor of cholangiocytes’ varied reaction to the immune-mediated damage. It indicates that the cholangiocytes actively participate in both innate and adaptive immune responses and constitute the biliary system’s first line of defense against foreign substances [57]. Likewise, the apical membrane of cholangiocytes is home to primary Cl^−^/HCO_3_^−^ anion exchanger 2 (AE2), which is in control of intracellular pH and biliary HCO_3_^−^ secretion and, as such, creates a bicarbonate-rich “umbrella” on its apical surface that shields them from harmful hydrophobic bile acids. Bile salts become hydrophobic and capable of passing through the plasma membrane when AE2 function is compromised, which triggers cellular death. Because soluble adenylyl cyclase (sAC) is a conserved bicarbonate sensor that makes cells more sensitive to apoptosis, downregulation of AE2 results in an alkaline intracellular environment [60]. Remarkably, large cholangiocytes are the primary cells that constitutively express the components needed for bicarbonate secretion. In particular, research has demonstrated that small cholangiocytes do not express secretin receptor (SR), cystic fibrosis transmembrane conductance regulator (CFTR), or AE2. Furthermore, only large bile ducts have elevated levels of cAMP, Cl^−^/HCO_3_^−^, AE2 activity, and ductal secretory activity in response to secretin stimulation [49]. Notably, a number of reports showed that PBC patients had discrepancies in the expression or control of the Cl^−^/HCO_3_^−^ exchanger and more recent studies indicated that a malfunctioning biliary bicarbonate “umbrella” on outer membrane of cholangiocytes may contribute to the development of some vanishing bile duct syndromes, including PBC [66,67]. The fact that PBC recurs following liver transplantation indicates that all cholangiocytes, including those from unaffected subjects, possess special biological characteristics that, under the correct circumstances, can cause the onset of autoimmune cholangitis [60].

## 10. Treatment of PBC

Ursodeoxycholic acid (UDCA), as an endogenous bile acid, has a variety of therapeutic effects, such as boosting bile acid enterohepatic circulation, stabilizing the biliary HCO_3_^−^ umbrella, preventing apoptosis, and reducing inflammation. It has been used to treat various liver diseases for more than a hundred years and it is still the usual first-line treatment for PBC. Despite the fact that UDCA monotherapy increased overall liver transplant-free survival, 30–40% of patients do not respond well to UDCA. According to a recent study, PBC patients benefited more from add-on therapy that combined UDCA with immunosuppressants or glucocorticoids [68]. When bezafibrate and UDCA were combined, the biochemical reaction was better and the anticipated mortality or liver transplantation requirement was lower than when UDCA was used alone.

Due to the disease’s specificity to AMA and to high blood immunoglobulin (Ig) M levels, it is indicated that B-cell-mediated processes are involved in PBC; therefore, targeting B-cells is a sensible therapeutic approach [69]. Rituximab is an anti-CD20 monoclonal antibody that selectively depletes B-cells. In PBC patients with an incomplete response to UDCA, rituximab treatment could improve alkaline phosphatase (ALP) levels, reduce the serum AMA titer, increase Treg cells numbers, and modulate cytokine production [68,70].

Bearing in mind that B-cell activating factor (BAFF) belongs to the TNF family and has a role in the pathogenesis of PBC, in a recent study, Zhang et al. showed that in the ARE-Del mice model of PBC, a combination of anti-BAFF and anti-CD20 decreased the number of B-cells, liver portal infiltration, and bile duct lesions [71].

## 11. Primary Sclerosing Cholangitis

Chronic fibrosing inflammation of the intrahepatic and/or extrahepatic biliary ducts is the histological hallmark of primary sclerosing cholangitis (PSC), a chronic, escalating cholestatic liver disease that eventually progresses to cirrhosis [72]. PSC meets the criteria for a rare disease, affecting fewer than 200,000 people in the United States and less than 5 in every 10,000 individuals in the European Union, with a total of under 250,000 cases across the EU [73]. Due to its rarity, gradual progression over many years, and the absence of reliable biomarkers for early detection, PSC poses significant challenges when it comes to the development of effective medical treatments [74]. Around 75% to 90% of people with PSC either have a previous diagnosis of or currently experience inflammatory bowel disease (IBD), with ulcerative colitis (UC) being the most common type [72]. Recent studies have indicated that the colitis linked to PSC is a distinct form of IBD, marked by a notably higher occurrence of rectal sparing and backwash ileitis [75]. In up to 25% of cases, individuals with PSC may also have other autoimmune conditions, such as type 1 diabetes, thyroid disorders, or rheumatoid arthritis. PSC predominantly affects males (about 65% of people with PSC are male) and most frequently occurs at around the age of 40. The epidemiological patterns of PSC and IBD support the theory that they are closely related, as they often coexist, have similar geographic prevalence, and IBD is found in the majority of PSC patients. While IBD is usually diagnosed before PSC, it can also develop after a diagnosis of PSC, even following a liver transplantation (LT) [76].

The majority of patients with PSC are asymptomatic at the time of diagnosis. In most cases, PSC is diagnosed incidentally when cholestasis is detected during screening of high-risk individuals, such as those with IBD or as part of preventive health checks [16,77]. The most common symptom among patients who exhibit symptoms is stomach discomfort (20%), which is followed by pruritus (10%), jaundice (6%), and exhaustion (6%). Importantly, 44% of PSC patients have hepatomegaly and 39% present with splenomegaly. However, there is a wide range of clinical manifestations of PSC [77]. The distinctive diagnostic sign of PSC is a cholangiogram presenting concentric fibrosis, with stenotic parts and pre-stenotic dilations of the intrahepatic or extrahepatic bile ducts, or both. These specific findings correspond to the image of “onion skin” lesions, the histological key feature of the disease, despite the fact they are found in less than 15% of affected individuals [16,77]. Although a liver biopsy does not usually detect these specific alterations, it is utilized to exclude overlapping syndromes and small-duct PSC, as well as to identify early biliary changes, and provides insights into the level of inflammation activity [78]. Despite its rarity, PSC is the leading cause of LT across Europe, as disease progression is unavoidable in the majority of patients, making LT the only life-saving option [79].

## 12. Pathogenesis of PSC

Although the exact pathogenesis of PSC has remained unclear for many years, several theories have been put forward. These include genetic, environmental, and immunological risk factors, as well as disruptions in the intestinal microbiota, the gut–liver axis, changes in bile composition, and alterations in the phenotype of BECs, all of which have been implicated in the development of PSC [79]. Cholangiocytes can be triggered by various factors, such as cholestasis, infections, ischemia, and xenobiotics. When activated, these cells exhibit increased proliferation and secrete pro-fibrotic and proinflammatory substances, which can ultimately result in biliary fibrosis and the development of cholangiocarcinoma [80]. One of the most recent and widely accepted etiopathogenetic theories is the PSC–microbiome hypothesis. This theory, although still evolving, builds upon the “leaky gut” hypothesis, which suggests that gut microbes (or their metabolic products) pass through a compromised gut barrier, often due to inflammation, and reach the liver through the hepatic portal system, where they initiate inflammatory and, eventually, fibrotic processes. This hypothesis, supported by evidence from in vitro studies, animal models, and human PSC cases, highlights several possible reasons for the development of PSC and is closely linked to the association between PSC and IBD. One factor is that weakening of the intestinal barrier increases the enterohepatic circulation of microbial byproducts. The second is associated with microbial dysbiosis, and the third refers to an abnormal, excessive cholangiocyte response to microbial molecules, particularly in the form of a senescence-associated secretory phenotype (SASP) [81].

Additionally, considering the gut microbiota is a metabolically highly active human organ, it is clear that changes in the diversity and composition of the gut microbiota play a role in the development of inflammatory and immune-mediated diseases, particularly those affecting the digestive and hepatobiliary systems, such as IBD and PSC. However, research has also shown that this influence extends to various autoimmune diseases, including diabetes, obesity, and atherosclerosis, and certain neurological disorders [82]. In comparison to healthy controls and patients with IBD alone, individuals with PSC show reduced microbial diversity and an overabundance of bacteria such as *Escherichia*, *Fusobacterium*, *Enterococcus, Lactobacillus*, *Blautia, Veillonella*, *Barnesiellaceae*, *Lachnospiraceae, Megasphaera*, *Rothia*, *Ruminococcus*, and *Streptococcus*. On the other hand, patients with PSC have lower levels of *Clostridium* cluster II, *Prevotella*, *Roseburia*, *Adlercreutzia*, and *Bacteroides* compared to both healthy individuals and those with IBD alone [83]. As a matter of fact, a cross-sectional cohort showed that one bacterial genus, *Veillonella*, was substantially overrepresented in people with PSC compared with both healthy controls and people with UC. A 4.8-fold increase was observed in people with PSC compared with healthy controls, and a 7.8-fold increase was observed in people with UC. In addition, this research revealed that the presence of IBD did not influence the gut microbiota in PSC patients. Furthermore, it was demonstrated that the reduction in gut microbiota diversity in PSC patients, which was the main finding of the study, was not associated with antibiotic use over the past 12 months [82]. It has been proven that episodes of bacterial infection (cholangitis) in patients with PSC may contribute to disease progression. Accordingly, recent studies suggest that pathogen-associated molecular patterns (PAMPs) could play significant roles in the development of both UC and PSC, since cholangiocytes constantly interact with them via their toll-like receptors (TLRs). It has been shown that innate immune responses to bacterial PAMPs in genetically predisposed rats cause portal inflammation and biliary strictures, resembling PSC. Additionally, the recruitment of gut-primed memory T-cells to the peribiliary space depends on the activation of cholangiocytes by bacterial PAMPs and proinflammatory cytokines. Another way the gut microbiota influences the pathogenesis of PSC and PSC-IBD is through the synthesis of secondary bile acids (BAs). Approximately 5% of primary BAs are deconjugated by bile salt hydrolases, enzymes produced by gut bacteria (such as *Clostridium*, *Bacteroides*, *Lactobacillus*, *Bifidobacterium*, and *Enterococcus* genus) in the distal ileum and colon, which leads to the formation of secondary BAs. The main secondary BAs are lithocholic acid (LCA) and deoxycholic acid (DCA), which can then be further altered by microorganisms to produce other secondary BAs. Bile acids act as signaling molecules, interacting with several receptors, including the nuclear Farnesoid X receptor (FXR) and the membrane-bound G protein-coupled bile acid receptor 1 (TGR5) [76]. The TGR5 (also referred to as GPBAR1) receptor is expressed throughout the biliary tree in BECs that line the small and large intrahepatic and extrahepatic ducts, as well as the gallbladder, in both humans and mice. TGR5 has been shown to have a protective role, as it helps produce chloride- and bicarbonate-rich bile secretion and influences the maintenance of tight junctions between BECs, thereby creating a protective barrier against toxic bile acids. Additionally, TGR5 positively influences the proliferation of BECs, which was shown to be decreased in Tgr5-/- mice in cholestasis models. Also, GWASs provide evidence that a genetic region linked to both ulcerative colitis and PSC includes the TGR5 gene locus. A previously conducted study compared the levels of TGR5 and cytokeratin 7 in PSC livers and healthy control livers, using antibody staining for both markers. The results revealed a significant reduction in TGR5 levels in both the early and late stages of the disease. Furthermore, to investigate the specific association between TGR5 and PSC and determine whether its downregulation is characteristic of PSC alone or whether it is common to other hepatobiliary diseases, an analysis was also performed on livers from patients with NASH, drug-induced liver injury (DILI), and chronic viral hepatitis. No reduction in TGR5 levels was observed in these liver samples, indicating that TGR5 downregulation is specific to PSC and may serve as an immunohistochemical marker for this disease. Moreover, in comparison with TGR5, no change in CK-7 intensity was observed in any of the analyzed livers. Also, the impact of IL8 on TGR5-expression was observed and stimulation of murine and human BECs with IL8/Il8 homologues resulted in decreased TGR5 mRNA and protein levels [79].

There are two hypotheses regarding the development of PSC—the autoimmune disease hypothesis (AID) and the immune-mediated inflammatory disease hypothesis (IMID). The difference between them lies in the proposed mechanisms: the AID hypothesis suggests that the loss of tolerance to a specific autoantigen is a key factor in the onset of the disease, while the IMID, on the other hand, attributes the disease to inadequate interaction between the innate and adaptive immune systems, which leads to persistent inflammatory infiltration in specific tissues, driven by the hyperproduction of proinflammatory mediators, primarily TNF-α. However, the AID has yet to identify a specific autoantigen, despite numerous candidates being proposed, and due to the inability to classify PSC as a typical autoimmune disease, some researchers consider it an atypical autoimmune disorder. Interestingly, although PSC was once considered an autoimmune disease, the classification of IBD as an IMID led to the inclusion of PSC in this category as well [75].

## 13. Genetic Factors

The involvement of genetic factors in the development of PSC is highlighted by the observation that first-degree relatives of individuals with PSC have a significantly higher risk of developing the disease, with an increase of up to 11 times [77]. It is clear that the vast number of genetic variants in the HLA complex region on chromosome 6 plays a crucial role in the pathogenesis of both autoimmune and immune-mediated diseases, including infections and disease susceptibility, with PSC being no exception. In the case of PSC, structural analysis of HLA class II suggests that HLA DRB1 may contain important information that can be used to identify peptide triggers. Additionally, the strong associations with HLA B further complicate efforts to outline the features of HLA DRB1 [84]. The strong association between HLA proteins and PSC has been demonstrated in numerous genetic studies, which have identified different alleles and HLA haplotypes that predispose individuals to PSC. These include HLA-DRB103, HLA-DRB113, HLA-B08:01, HLA-DQB102, and HLA-DQA105:01. On the other hand, protective alleles such as HLA-DRB104 and HLA-DQB1*03:02 have also been identified. All of these findings highlight the importance of acquired immunity in the onset and progression of PSC [85]. Once the numerous challenges associated with analyzing and interpreting HLA findings in PSC are addressed, the insights gained will likely reveal key antigenic triggers, and possibly even causal ones. Moreover, non-HLA genes within the HLA region are likely to increase the autoimmune disease risk, too. To date, 22 non-HLA susceptibility loci for PSC have been identified, each showing genome-wide statistical significance. However, the exact significance of their contribution remains unclear [84].

## 14. PSC-IBD Association

Given the frequent coexistence of PSC and IBD, there is a growing need to investigate the shared factors that promote their development in order to better understand the pathogenesis of IBD-PSC. In this context, it has been found that both the innate and adaptive immune systems are affected. A previously noted connection between PAMP molecules from intestinal bacteria and TLR signaling proteins on cholangiocytes has been further explored. A characteristic of PSC patients is the increased expression of TLRs and activation of myeloid differentiation factor 88 (MyD88), which results in pathological activity of the innate immune system and the hyperproduction of proinflammatory mediators such as IL-1α, IL-6, and monocyte chemotactic protein-1 (MCP-1/CCL2), which promote chronic inflammatory changes, leading to fibrosis. Additionally, the disruption of CFTR protein regulation, which affects bicarbonate secretion, has also been implicated in the pathogenesis of biliary tree diseases. Moreover, the PSC-IBD phenotype is proven to be characterized by excessive infiltration of T lymphocytes in both the colon and liver. Also, the presence of certain serological markers, such as pANCA autoantibodies, which are common in both UC and PSC, supports the idea of an abnormal immune response to intestinal antigens, which appears to be a common and potentially crucial pathogenic factor in both diseases and their overlapping pathways [85].

## 15. A Closer Look into Existing Medical Treatments and Future Treatment Modalities

Although LT may serve as a life-saving intervention, it is not a guaranteed cure, as there are documented cases of disease recurrence occurring several years post-transplant [73]. Additionally, no medical treatment has been proven to modify the progression of PSC. The existing approaches primarily aim to manage symptoms and treat complications such as bacterial cholangitis and comorbidities like IBD or other autoimmune and metabolic disorders [77]. For example, UDCA is a naturally derived bile acid that is commonly used to manage PSC (thus, it is also used to treat related cholestatic disorders such as PBC), despite having no real impact on survival rates, the incidence of LT, or the development of cholangiocarcinoma. Nevertheless, various studies have reported reductions in serum bilirubin, ALT, and ALP levels in response to different doses of UDCA (10–15 mg, 13–15 mg, 17–23 mg) [74]. Surprisingly, several large-scale studies that investigated the effects of high doses of UDCA (28–30 mg/kg/day) were terminated prematurely due to an unexpected 2.3-fold increase in the risk of disease progression to liver transplantation (LT) and/or the development of varices in the treatment group, despite a statistically significant improvement in liver biochemistry. It was later found that high doses of UDCA may contribute to adverse outcomes, such as an increased risk of colorectal cancer, hepatobiliary malignancies, and liver decompensation, necessitating a liver transplant or, in some cases, leading to death [86]. Despite this, considering the fact that when used in low-to-moderate doses, UDCA positively affects bicarbonate secretion, biliary apoptosis, and hepatic biochemistry, it continues to be used as a first-line therapy for PSC [87]. Current guidelines recommend the prophylactic use of antibiotics in patients with recurrent bacterial cholangitis (a sign of advanced PSC that may accelerate its progression) and those undergoing biliary interventions. Additionally, several small studies on the use of antibiotics in people with PSC have shown promising results, evaluating the efficacy of both low and high doses of metronidazole and vancomycin in improving liver biochemistry, inflammatory markers, and symptoms. Future treatments, including fecal and bile transplantation that can modify the microbiome, may play a key role in managing PSC [88]. Furthermore, obstructions of the bile ducts, which originate from periductal fibrosis, the primary characteristic of PSC, are treated endoscopically—through balloon dilation or stent placement—or, in some cases, surgically. New therapies are being investigated, including immunosuppressive drugs (glucocorticoids, azathioprine, ciclosporin, tacrolimus, and methotrexate) and biologics. These are still in the research phase.

Given the inherent complexity of the etiopathogenesis of PSC, it is understandable that, despite decades of research, an effective causal therapy remains elusive. As a result, numerous ongoing trials and research studies are focused on exploring the potential of future treatments that target the immune-mediated core of the disease. For example, treatments such as 24-Nor-ursodeoxycholic acid (NorUDCA), lysyl-oxidase 2 (LOXL2) inhibitors, vedolizumab, all-trans retinoic acid (ATRA), and obeticholic acid, an FXR agonist, have shown promising results.

## 16. Conclusions

Although numerous medical researchers have endeavored to elucidate the exact pathophysiological mechanisms of AILD, the exact pathways have yet to be determined and remain incompletely understood. Recent advances in our understanding of key points in these mechanisms bring hope that therapeutic modalities will reach their full potential, offering immunomodulatory benefits, protective effects on the liver and biliary tract, and the ability to slow progression, ultimately preventing these diseases from advancing to their end-stage forms.
